# Ultrasonographic diaphragmatic assessment as an emergency mortality predictor in acute exacerbation of COPD

**DOI:** 10.1186/s12245-026-01130-3

**Published:** 2026-02-19

**Authors:** Athar Fekry Lasheen, Sami Sayed Ahmed El-dahdoh, Mahmoud Tarek AbdElsamea Gadallah, Hatem Mahmmoud Sultan

**Affiliations:** 1 Emergency Medicine, Faculty of Medicine, Menoufia University, Shebin Elkom, Egypt; 2 Chest Disease and Tuberculosis, Faculty of Medicine, Menoufia University, Shebin Elkom, Egypt; 3 General Surgery, Faculty of Medicine, Menoufia University, Shebin Elkom, Egypt; 4 GIT and Laparoscopic Surgery, Faculty of Medicine, Menoufia University, Shebin Elkom, Egypt

**Keywords:** COPD, Diaphragm, POCUS, Emergency, Mortality

## Abstract

**Objectives:**

To evaluate the role of diaphragmatic ultrasound as a mortality predictor in acute exacerbation of chronic obstructive pulmonary disease (AECOPD), compared to the DECAF and BAP65 scoring systems.

**Background:**

The diaphragm is the primary muscle of respiration and is often compromised during AECOPD. Bedside ultrasound offers a non-invasive method to assess diaphragmatic function. Early identification of patients at high risk of mortality upon presentation may aid in appropriate triage and management.

**Methods:**

This observational pilot study included 50 patients presenting to the emergency department with AECOPD. For each patient, the BAP65 score (Blood urea nitrogen, altered mental status, Pulse, Age ≥ 65), DECAF score (Dyspnea, Eosinopenia, Consolidation, Acidemia, Atrial fibrillation), diaphragmatic thickness fraction (diTF), and diaphragmatic excursion (diEX) were recorded at presentation.

**Results:**

Of the 63 patients initially enrolled, 50 completed the study, with a predominance of male participants. Mortality occurred in 16% of cases. Deceased patients were significantly more likely to have comorbidities such as diabetes mellitus, hypertension, atrial fibrillation, ischemic heart disease, cardiomyopathy, cerebrovascular stroke, chronic kidney disease, and reliance on home oxygen therapy (*P* < 0.05). Receiver operating characteristic (ROC) analysis identified cutoff values for each predictor: BAP65 ≥ 5, DECAF ≥ 3, diTF ≤ 21%, and diEX ≤ 2.5 cm. Corresponding area under the curve (AUC) values were: BAP65, 0.833, CI = 0.621 to 1.000; DECAF, 0.976, CI = 0.933 to 1.000; diTF, 0.848, CI = 0.670 to 1.026; and diEX, 0.884, CI = 0.787 to 0.981. Spearman’s rank correlation revealed a strong negative correlation between diEX and DECAF (*r* = -0.771, CI= -0.862–-0.628, *p* < 0.001), and between diEX and BAP65 (*r* = -0.626, CI = -0.757–-0.448, *p* < 0.001). Moderate negative correlation was also observed between diTF and DECAF (*r* = -0.451, CI = 0.616–-0.241, *p* < 0.001), and weak negative correlation between diTF and BAP65 (*r* = -0.341, CI =-0.570–-0.069, *p* = 0.01). However, these finding should be interpreted with caution due to small sample size.

**Conclusion:**

Diaphragmatic ultrasound is a rapid and reliable tool for predicting mortality in patients with AECOPD presenting to the emergency department. It demonstrates comparable or superior predictive value to established scoring systems such as BAP65 and DECAF. However, this finding should be interpreted with caution and further studies with larger sample sizes are warranted to validate these findings.

## Introduction

 Acute exacerbation of chronic obstructive lung disease (AECOPD) has recently been updated as an event characterized by dyspnea and/or cough with sputum that worsens within 14 days, which may be accompanied by tachypnea and/or tachycardia and is often associated with increased local and systemic inflammation caused by airway infection, pollution, or another insult to the airways [1].

COPD is the fourth leading cause of death worldwide, accounting for 3.5 million deaths in 2021 and about 5% of all deaths [2]. Most deceased patients were males under the age of 70 years, occurring in low- and middle-income countries [3]. Moreover, hospitalization due to a COPD exacerbation has a poor long-term prognosis, with a five-year mortality rate of almost 50% [4, 5].

The BAP65 and DECAF scoring systems are well-known systems for assessing the severity and mortality of COPD exacerbations [6–8]. The BAP65 stands for blood urea nitrogen, altered mental status, pulse, and age ≥ 65 years. Additionally, the DECAF stands for dyspnea, eosinopenia, consolidation, acidemia, and atrial fibrillation [9]. The BAP65 and DECAF scores were significantly associated with non-invasive ventilation (NIV) failure and higher mortality in AECOPD, but they required more time to assess the prognosis of AECOPD patients [8].

The diaphragm is the principal muscle of inspiration, generating negative intrathoracic pressure. During AECOPD, hyperinflation of both lungs impairs the ability to generate trans-diaphragmatic negative intrathoracic pressure [1, 10]. The gold standard method for evaluating diaphragmatic function is measuring the negative pressure produced during muscle contraction. Moreover, diaphragmatic dysfunction, which is frequently observed in AECOPD, has been associated to non-invasive failure and increased mortality rates [11–14]. Ultrasound-based assessment of diaphragmatic thickness fraction (diTF) and excursion (diEX) has been proposed as a reliable and practical method for predicting weaning failure in intensive care settings [13].

Although BAP-65 and DECAF are considered reliable tools in prediction mortality, their applicability is not du=dynamic and cannot assess the progress or deterioration after management. Moreover, both need long period of time to make a conclusion. We conducted this study to assess the role of diaphragmatic ultrasound (diTF, diEX) as a predictor of mortality among AECOPD patients and compare the finding with DECAF/BAP65 scores.

## Methods

We conducted a pilot observational study involving 50 patients who presented to the Emergency Department of the Faculty of Medicine, Menoufia University, between April 2023 and December 2024. The study was performed in accordance with the Declaration of Helsinki. Written informed consent was obtained from all participants or their first-degree relatives prior to data collection; patients who declined consent were excluded. The study protocol was approved by the Ethics Committee of the Faculty of Medicine, Menoufia University.

Patients aged over 40 years presenting with acute exacerbation of chronic obstructive pulmonary disease (AECOPD) were eligible. Exclusion criteria included the need for endotracheal intubation upon presentation (due to the urgent requirement to secure the airway and maintain oxygenation/ventilation, which precluded standardized ultrasound), phrenic nerve palsy, neuromuscular disease, prior chemical pleurodesis, chest wall deformities, interstitial lung disease, or pregnancy. Patients requiring non-invasive ventilation were included.

All participants underwent detailed history taking, including age, sex, smoking status, presenting complaints (shortness of breath, cough), comorbidities (diabetes mellitus, hypertension, atrial fibrillation, ischemic heart disease, cardiomyopathy, stroke, chronic liver disease, renal disease), and oxygen requirements. Clinical assessment included vital signs (respiratory rate, heart rate, oxygen saturation, systolic and diastolic blood pressure), general examination (Glasgow Coma Scale, cyanosis, clubbing, lower limb edema, hepatomegaly), respiratory system examination (inspection, palpation, percussion, auscultation), the Extended Medical Research Council Dyspnea Scale, and severity scores (BAP65 and DECAF).

Diaphragmatic function was assessed using the Butterfly iQ device (Lightning, 2019; Butterfly Network, Inc., Guilford, Connecticut, USA) by an experienced operator. M-mode ultrasound was employed to measure diaphragmatic thickness and excursion. Diaphragmatic Excursion (diEX): Measured at the right mid-clavicular line below the costal margin through the hepatic window. Normal lower limits for maximal inspiration were 3.7 cm in women and 4.7 cm in men [15]. Diaphragmatic Thickness Fraction (diTF): Calculated as the difference between inspiratory and expiratory diaphragmatic thickness divided by expiratory thickness, expressed as a percentage [16]. Measurements were taken at the right mid-axillary line within the zone of apposition (8th–10th intercostal spaces). Normal values were 1.7 ± 0.2 mm in expiration and 4.5 ± 0.9 mm in deep inspiration. A diTF < 20% was considered indicative of diaphragmatic dysfunction [11].

Diaphragmatic assessment in patients with AECOPD was performed by an emergency medicine physician with approximately ten years of experience in point-of-care ultrasound (POCUS) within the emergency setting. The operator had undergone specialized training in diaphragmatic evaluation under the supervision of a senior faculty member at the same institution, with particular emphasis on lung and diaphragmatic assessment. This structured training spanned three months, during which the operator conducted more than 100 diaphragmatic examinations. For the purposes of this study, the operator was blinded to all clinical and investigational data of the enrolled AECOPD patients.

Patients were monitored throughout their hospital stay, whether in the Emergency Department, Chest Ward, or Intensive Care Unit. Outcomes were categorized as either discharged alive or deceased.

Data were analyzed using IBM SPSS Statistics for Windows, version 28.0 (IBM Corp., Armonk, NY, USA; released in 2021). Descriptive statistics summarized demographic, clinical, and investigational characteristics. Continuous variables were presented as mean ± standard deviation (SD) for normally distributed data, or as median and interquartile range (IQR) for non-normally distributed data. Additionally, categorical variables were expressed as frequencies and percentages.

Comparative analyses were performed as follows: Fisher’s Exact Test was used for categorical variables, Independent Samples T-test was applied to compare normally distributed continuous variables, and Mann–Whitney U Test was used for non-normally distributed continuous variables. Normality of continuous variables was assessed using the Shapiro–Wilk test and by visual inspection of histograms and Q–Q plots.

Receiver operating characteristic (ROC) curve analysis was performed to evaluate the diagnostic performance of selected predictors. The area under the curve (AUC), sensitivity, specificity, positive predictive value (PPV), negative predictive value (NPV), and overall accuracy were reported. Optimal cut-off values were determined using the Youden index, maximizing sensitivity and specificity.

Spearman’s rank correlation coefficient was used to assess associations between BAP65, DECAF scores, diaphragmatic thickness fraction (diTF), and diaphragmatic excursion (diEX). All statistical tests were two-sided, with *p* < 0.05 considered statistically significant.

## Results

Out of 63 screened patients with acute exacerbation of chronic obstructive pulmonary disease (AECOPD), 13 were excluded based on the study’s inclusion and exclusion criteria. The remaining 50 patients were enrolled. Among them, 42 patients were discharged alive, while 8 patients died following treatment—either in the emergency department, hospital ward, or intensive care unit (ICU).

Most patients were male (80%), older than 65 years (58%), smokers (100%), and presented with shortness of breath (100%) and productive cough (44%). The most common comorbidities among the included participants were atrial fibrillation (52%), diabetes mellitus (40%), and hypertension (40%).

There was no statistically significant difference in mortality between male and female patients. The median age of discharged patients was 65 years, compared to 70 years in the deceased group. Mortality was significantly higher among patients older than 65 years (*P* = 0.015).

Several comorbidities were significantly more prevalent in the deceased group compared to those discharged, including: Diabetes mellitus (*P* = 0.047), Hypertension (*P* = 0.005), Atrial fibrillation (*P* = 0.004), Ischemic heart disease (*P* = 0.009), Cardiomyopathy (*P* = 0.041), Cerebrovascular stroke (*P* = 0.011), Chronic kidney disease (*P* = 0.001), Home oxygen therapy (*P* < 0.001) as shown in Table 1.

**Table 1:** Socio-demographic characteristics, comorbidities, and presenting complaints in alive and dead hospitalized AECOPD patients

Variable	Alive (*N* = 42)	Died (*N* = 8)	*P*-value
**Socio-demographic data**			
Sex, n (%)			
Male	33 (78.6%)	7 (87.5%)	1.00
Female	9 (21.4%)	1 (12.5%)	
Age Categories, n (%)			
45–65	21 (50%)	0	0.015
> 65	21 (50%)	8 (100%)	
Age, Median (IQR)	65 (18)	70 (11)	0.39
Smoking Exposure	42(100%)	8(100%)	
**Presenting complaints**, n (%)			
Shortness of Breath	40 (95.2%)	8 (100%)	1.00
Cough & Expectoration	20 (47.6%)	2 (25%)	0.439
**Comorbidities**, n (%)			
Diabetes Mellitus, n (%)	14 (33.3%)	6 (75%)	0.047
Hypertension, n (%)	13 (31%)	7 (87.5%)	0.005
Atrial Fibrillation	18 (42.9%)	8 (100%)	0.004
**Ischemic Heart Disease**	3 (7.1%)	4 (50%)	0.009
Cardiomyopathy	6 (14.3%)	4 (50%)	0.041
Cerebrovascular Stroke	1 (2.4%)	3 (37.5%)	0.011
Chronic Liver Disease	9 (21.4%)	2 (25%)	1
Chronic Kidney Disease	3 (7.1%)	5 (62.5%)	0.001
O₂ treatment	5 (11.9%)	7 (87.5%)	< 0.001
extended Medical Research Council of Dyspnea (eMRCD), n (%)			
< eMRCD 5, n (%)	40 (95.2%)	1 (12.5%)	< 0.001
eMRCD 5a, n (%)	2 (4.8%)	7 (87.5%)	
eMRCD 5b, n (%)	0	0	
**Vital signs examinations**			
Respiratory Rate (RR), Mean ± SD	25.7 ± 7.4	35.6 ± 10.4	0.002
Oxygen Saturation (SpO₂), Median (IQR)	85 (10)	71 (21)	0.001
Heart Rate (HR) (bpm), Mean ± SD	104.8 ± 18.4	124.9 ± 14.2	0.005
HR ≥ 109, n (%)	18 (42.9%)	7 (87.5%)	0.049
Systolic BP, Median (IQR)	120 (20)	90 (25)	0.047
Diastolic BP, Median (IQR)	70 (10)	60 (17.50)	0.074
**General physical examination**			
Glasgow Coma Scale (GCS), Median (IQR)	15 (0)	12.50 (2)	< 0.001
Altered sensorium (GCS < 14), n (%)	4 (9.5%)	8 (100%)	< 0.001
Cyanosis, n (%)	5 (11.9%)	6 (75%)	< 0.001
Clubbing, n (%)	5 (11.9%)	6 (75%)	< 0.001
Lower limb Oedema, n (%)	9 (21.4%)	7 (87.5%)	< 0.001
Enlarged Tender Liver, n (%)	6 (14.3%)	6 (75%)	0.001
**Respiratory system examination**, n (%)			
Inspection (Hyper inflated), n (%)	42 (100%)	8 (100%)	-
Palpation (Tenderness), n (%)	0	1 (12.5%)	0.160
Percussion (Dull), n (%)	6 (14.3%)	8 (100%)	< 0.001
Auscultation (Wheezes), n (%)	39 (92.9%)	7 (87.5%)	0.514
Auscultation (Crepitation), n (%)	16 (38.1%)	8 (100%)	0.001

Continuous variables are presented as mean ± SD for normally distributed data and as median (IQR) for non-normally distributed data.

Vital signs in the deceased group showed significantly higher respiratory and heart rates (*P* = 0.002 and *P* = 0.005), lower oxygen saturation (*P* = 0.001), tachycardia > 109 bpm (*P* = 0.049), and reduced systolic blood pressure (*P* = 0.047). Clinical signs such as altered mental status (GCS < 14), cyanosis, clubbing, and lower limb edema were also more frequent (*P* < 0.001) as shown in Table 1.

Most patients presented with shortness of breath or changes in cough and expectoration. Dyspnea severity over the past three months, assessed by the eMRCD scale, was significantly worse in the deceased group, especially class 5a (*P* < 0.001). Chest exam revealed more dullness on percussion (*P* < 0.001) and crepitations (*P* = 0.001), while hyperinflation, tenderness, and wheezing showed no significant difference.

Arterial blood gases indicated more acidemia (pH < 7.35) and hypercapnia (CO₂ > 45 mmHg) in the deceased group (*P* < 0.001). Lab findings included higher rates of leukocytosis, eosinopenia (both *P* < 0.001), elevated BUN (*P* = 0.001), sodium (*P* = 0.005), potassium (*P* = 0.034), and D-dimer (*P* < 0.001).

Chest consolidation and atrial fibrillation were present in all deceased patients, compared to 57% and 43% in the discharged group (*P* = 0.039 and *P* = 0.004). Right and left cardiac impairments were also significantly more common in the deceased group (*P* < 0.001), as shown in Table 1.

There was a statistically significant response to medical treatment and noninvasive ventilation (NIV) among patients who survived (*P* = 0.005 and *P* = 0.013, respectively). In contrast, the need for invasive mechanical ventilation was significantly higher in the deceased group (*P* < 0.001).

Significant differences were also observed between survivors and non-survivors in terms of prognostic scores and diaphragmatic ultrasound parameters recorded upon emergency department presentation. Median values were as follows: BAP65 score (3 vs. 5, *P* = 0.002), DECAF score (2 vs. 5, *P* < 0.001), diaphragmatic thickness fraction (diTF: 37.5% vs. 15.6%, *P* = 0.001), and diaphragmatic excursion (diEX: 3.4 cm vs. 1.7 cm, *P* < 0.001), as shown in Table 2.

**Table 2:** Mortality prediction scores between alive and died hospitalized patients with AECOPD

Variable	Alive (*N* = 42)	Died (*N* = 8)	*P*-value
BAP65 score, Median (IQR)	3 (1)	5 (2)	0.002
DECAF score, Median IQR	2 (1)	5 (1)	< 0.001
**Diaphragmatic thickness**			
Thickness of diaphragm in expiration, Mean ± SD	1.8 ± 0.6	1.6 ± 0.7	0.460
Thickness of diaphragm in inspiration, Mean ± SD	2.5 ± 1.0	1.9 ± 0.8	0.085
Thickness Fraction ( DiTF) (%), Median (IQR)	0.375 (0.35)	0.156 (0.24)	0.001
**Diaphragmatic Excursion**			
Diaphragmatic Excursion ( DiEX), Median (IQR)	3.4 (2.2)	1.7 (0.5)	< 0.001

**Fig. 1:**
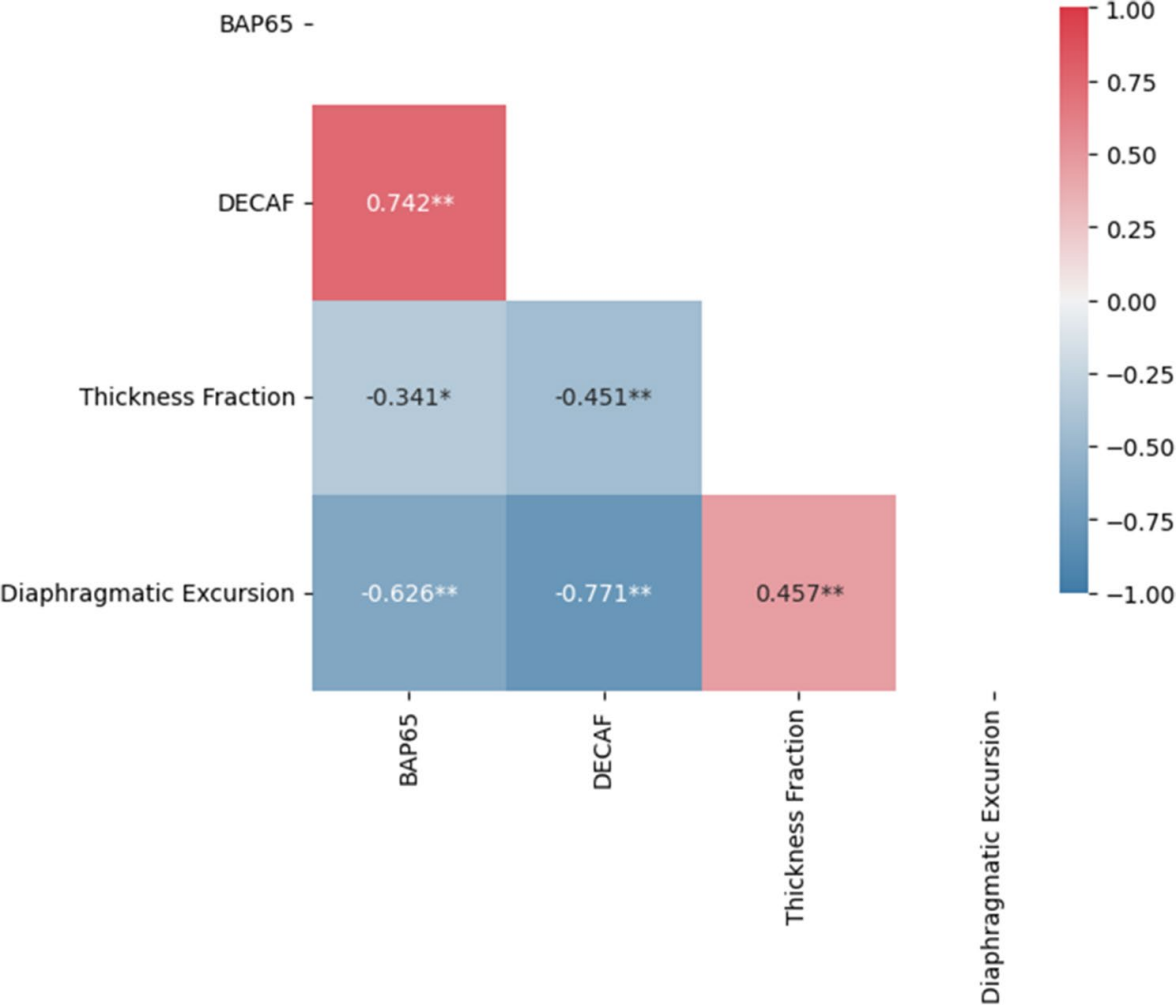
Heatmap of correlations between BAP65, DECAF, thickness fraction, and diaphragmatic excursion

Correlation analysis, illustrated in a heat map, showed a strong negative correlation between diaphragmatic excursion and both DECAF score (*r* = − 0.771, CI = -0.862–-0.628, *p* < 0.001) and BAP65 score (*r* = − 0.626, CI = -0.757–-0.448, *p* < 0.001). Diaphragmatic thickness fraction also demonstrated a moderate negative correlation with DECAF (*r* = − 0.451, CI = -0.616–-0.241, *p* < 0.001) and weak negative correlation for BAP65 (*r* = − 0.341, CI = -0.570–-0.069, *p* = 0.01). A strong positive correlation was found between BAP65 and DECAF scores (*r* = 0.742, CI = 0.589–0.845, *p* < 0.001), while a moderate positive correlation was observed between diTF and diEX (*r* = 0.457, CI = 0.245–0.619, *p* < 0.001) as shown in Fig. 1; Table 3.

**Table 3:** Exploratory correlation matrix of prognostic predictors in hospitalized AECOPD patients

Variables	BAP65	DECAF	Thickness Fraction	Diaphragmatic Excursion
BAP65	1	0.742 (0.589–0.845), *p* < 0.001	-0.341 (-0.570–-0.069), *p* = 0.01	-0.626 (-0.757–-0.448), *p* < 0.001
DECAF	0.742 (0.589–0.845), *p* < 0.001	1	-0.451 (-0.616–-0.241), *p* < 0.001	-0.771 (-0.862–-0.628), *p* < 0.001
Thickness Fraction	-0.341 (-0.570–-0.069), *p* = 0.01	-0.451 (-0.616–-0.241), *p* < 0.001	1	0.457 (0.245–0.619), *p* < 0.001
Diaphragmatic Excursion	-0.626 (-0.757–-0.448), *p* < 0.001	-0.771 (-0.862–-0.628), *p* < 0.001	0.457 (0.245–0.619), *p* < 0.001	1

Correlations were assessed using Spearman’s rank correlation coefficient.

Correlation analyses are exploratory and reflect relationships among predictors rather than direct associations with mortality.

Receiver Operating Characteristic (ROC) curve analysis identified cutoff points for mortality prediction: BAP65 ≥ 5, DECAF ≥ 3, diTF ≤ 21%, and diEX ≤ 2.5 cm. These thresholds were associated with corresponding measures of accuracy, sensitivity, specificity, and predictive values, detailed in Table 4.

**Table 4:** Validity of BAP65, DECAF, diaphragmatic thickness fraction and excursion in predicting of mortality in hospitalized patients with AECOPD

**Prediction of Mortality**	**BAP65 Score**		**DECAF Score**	
Best cutoff point	≥ 5		≥ 3	
AUC (95% CI)	0.833	0.621 to 1.000	0.976	0.933 to 1.000
Sensitivity	75%	34.91% to 96.81%	100%	63.06% to 100.00%
Specificity	97.62%	87.43% to 99.94%	76%	60.55% to 87.95%
Positive Predictive Value	86%	45.37% to 97.74%	44%	32% to 58%
Negative Predictive Value	95%	86.05% to 98.55%	100%	89% to 100%
Accuracy	94%	83.45% to 98.75%	80%	66.28% to 89.97%
	**Thickness Fraction**		**Diaphragmatic Excursion**	
Best cutoff point	≤ 21%		≤ 2.5 cm	
AUC (95% CI)	0.848	0.670 to 1.026	0.884	0.787 to 0.981
Sensitivity	88%	47.35% to 99.68%	100%	63.06% to 100.00%
Specificity	86%	71.46% to 94.57%	71%	55.42% to 84.28%
Positive Predictive Value	54%	34.71% to 71.91%	40%	29.24% to 51.82%
Negative Predictive Value	97%	85.14% to 99.56%	100%	88.43% to 100.00%
Accuracy	86%	73.26% to 94.18%	76%	61.83% to 86.94%

## Discussion

The mortality rate of acute exacerbations of COPD (AECOPD) varies across studies. In our cohort, mortality was 16%, compared to 4% in Shorr et al. [17], 10.4% in Steer et al. [7], and 18% in Sangwan et al. [6]. Such differences may reflect variations in admission thresholds and healthcare systems across countries [3].

Mortality was significantly higher in patients aged > 65 years, likely due to comorbidities, recurrent exacerbations, and age-related lung changes [10]. Smoking history, measured by pack-years and duration, also influenced disease severity [18]. While Zhang et al. reported similar survival between sexes, female smokers had worse in-hospital outcomes [19].

Comorbidities including diabetes, hypertension, atrial fibrillation, ischemic cardiomyopathy, cerebrovascular stroke, and chronic kidney disease were strongly associated with mortality, consistent with prior studies [20, 21]. Lin et al. [22] highlighted that patient with type 2 diabetes compared to non-diabetic patients showed lower oxygen levels, longer hospital stays, and higher mortality. Cardiovascular disease was similarly linked to prolonged hospitalization, mechanical ventilation, and increased mortality [23]. Pulmonary hypertension [21] and atrial fibrillation risk factors such as age, uric acid, and left atrial diameter further contributed to poor outcomes [24].

Clinical signs—tachypnea, hypoxia, tachycardia, hypotension, altered mental status, cyanosis, clubbing, and edema—were more common in deceased patients. These features, also emphasized by Zhou et al. [25] and Pahal et al. [18], reflect severe hypoxia, polycythemia, and consolidation, and were predictive of poor prognosis [26].

Hypercapnia and respiratory acidosis were significantly associated with mortality, in line with Trethewey et al. [27], who identified acid-base disturbances as critical prognostic factors. Hematological abnormalities such as leukocytosis and eosinopenia also predicted mortality, consistent with recent evidence supporting eosinopenia, lymphocytopenia, and elevated neutrophil-to-lymphocyte ratio as strong prognostic markers [28, 29].

Renal impairment, with elevated potassium, sodium, and urea, was more frequent among deceased patients. Impaired bicarbonate reabsorption during exacerbations may worsen respiratory acidosis, leading to mixed acidosis and higher mortality [18].

Recently published literature highlighted the impact of using validated scoring systems in predicating mortality among AECOPD patients in emergency department. BAP-65 (blood urea nitrogen, altered mental status, pulse ≥ 109/min, and age ≥ 65 years) has been predicted the in-hospital mortality, particularly in resource-limited settings [9, 30]. Although our finding showed similar findings, these findings should be interpreted with caution due to the exploratory nature of our research and small sample size.

Additionally, DECAF score (dyspnea severity, eosinopenia, consolidation, acidemia, and atrial fibrillation) is also used for predicting the in-hospital mortality of AECOPD patients. A recently published study comparing BAP-65, DECAF, and other scoring systems showed that DECAF had the highest ability in predicting the mortality [30, 31]. However, this needs a lot of time for assessing these patients in the emergency department. Moreover, scoring systems are not a dynamic tool for assessing the improvement of deterioration of AECOPD at the emergency department.

Evidence shows that diaphragmatic ultrasound has a significant prognostic role in acute exacerbations of COPD (AECOPD), particularly in predicting mortality [32, 33]. Diaphragmatic dysfunction—seen as reduced excursion and thickening fraction—is a marker of respiratory muscle fatigue and impending ventilatory failure [32]. Both prospective and retrospective studies link these abnormalities to prolonged mechanical ventilation and higher mortality [11, 16, 32, 33].

Ultrasound offers a non-invasive, bedside, rapid, and repeatable way to assess diaphragmatic function, giving clinicians an early warning of patients at risk of deterioration. Recent multicenter data confirm that ultrasound parameters independently predict in-hospital mortality, highlighting its value in guiding escalation of care and resource allocation [34]. These finding was also supported by our finding. However, due to the exploratory nature of our manuscript, these finding should be taken with cautious.

Moreover, when assessing the correlation between diaphragmatic ultrasound and BAP-65 and DECAF. Diaphragmatic excursion showed strong negative correlation with DECAF and BAP-, Moderate negative correlation was also observed between diTF and DECAF, and weak negative correlation between diTF and BAP65. Additionally, when adding the diaphragmatic ultrasound with established scoring systems, including BAP-65 and DECAF this can improve risk stratification of AECOPD patients.

Point-of-care ultrasound (POCUS) provides immediate bedside assessment in acute exacerbations of COPD, offering advantages over traditional scoring systems. Although the DECAF score demonstrates high predictive accuracy (AUC = 0.976), its use depends on laboratory results that may delay decision-making in the emergency department. In contrast, diaphragmatic ultrasound—measuring excursion and thickening fraction—delivers real-time physiological data by directly visualizing diaphragmatic function and identifying mechanical failure due to hyperinflation. This enables rapid triage, with high-risk patients recognized within minutes, and allows dynamic monitoring through serial evaluations to assess response to non-invasive ventilation or progression of fatigue. Diaphragmatic indices should therefore be regarded not as replacements for validated scores, but as complementary tools that enhance immediate clinical decision-making and support early escalation of care.

This study has several limitations that should be acknowledged. First, small sample size of 50 patients with AECOPD could influence the robustness of the conclusions. Second, the small number of mortality events precluded the use of multivariable regression analysis, restricting our ability to adjust for potential confounders; therefore, the results should be interpreted with caution. Third, intubated patients were excluded, which may affect the generalizability of the findings to more severe cases. it remains unclear whether patients younger than 40 years presenting with AECOPD were excluded, and if so, this may introduce bias in assessing the association between age and mortality. Finally, most group comparisons were primarily reported using p-values without corresponding effect size estimates or confidence intervals, which may limit the clinical interpretability of the findings. Although confidence intervals were provided for ROC analyses, future larger-scale studies are encouraged to report comprehensive effect size measures. Future studies with larger, more diverse cohorts are warranted to validate these results and enable more comprehensive multivariable modeling.

## Conclusion

Our exploratory research article showed that diaphragmatic ultrasound can be considered a reliable and rapid tool for assessment of AECOPD patient compared to BAP65 and DECAF scoring system. Diaphragmatic indices should therefore be regarded not as replacements for validated scores, but as complementary tools that enhance immediate clinical decision-making. Moreover, future research articles with larger sample size are warranted to validate these finding.

## Data Availability

All data used in this study is available upon request.
